# Molecular Generative Model via Retrosynthetically Prepared Chemical Building Block Assembly

**DOI:** 10.1002/advs.202206674

**Published:** 2023-01-03

**Authors:** Seonghwan Seo, Jaechang Lim, Woo Youn Kim

**Affiliations:** ^1^ HITS Incorporation 124 Teheran‐ro, Gangnam‐gu Seoul 06234 Republic of Korea; ^2^ Department of Chemistry KAIST, 291 Daehak‐ro, Yuseong‐gu Daejeon 34141 Republic of Korea; ^3^ AI Institute KAIST, 291 Daehak‐ro, Yuseong‐gu Daejeon 34141 Republic of Korea

**Keywords:** deep generative model, deep learning, molecular design, synthesizability

## Abstract

Deep generative models are attracting attention as a smart molecular design strategy. However, previous models often render molecules with low synthesizability, hindering their real‐world applications. Here, a novel graph‐based conditional generative model which makes molecules by tailoring retrosynthetically prepared chemical building blocks until achieving target properties in an auto‐regressive fashion is proposed. This strategy improves the synthesizability and property control of the resulting molecules and also helps learn how to select appropriate building blocks and bind them together to achieve target properties. By applying a negative sampling method to the selection process of building blocks, this model overcame a critical limitation of previous fragment‐based models, which can only use molecules from the training set during generation. As a result, the model works equally well with unseen building blocks without sacrificing computational efficiency. It is demonstrated that the model can generate potential inhibitors with high docking scores against the 3CL protease of SARS‐COV‐2.

## Graphical abstract

The authors propose a novel generative model that assembles retrosynthetically prepared building blocks to improve the synthesizability. The model can efficiently utilize dozens of thousands of building blocks for molecule generation, which was not possible in previous models. They demonstrate the applicability of the model in inhibitor design by generating molecules with high binding scores against SARS‐COV‐2.

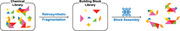

## 1 Introduction

The design of molecules with desired properties is at the heart of chemistry. Deep learning‐based molecular design has attracted great attention as a new strategy for various applications including drug design.^[^ ^1^, ^2^, ^3^, ^4^, ^5^ ^]^ The so‐called deep generative models aim to precisely control multiple properties at the same time while navigating the vast chemical space. That becomes possible by learning the structure–property relationships directly from raw data implying both structure and property information on diverse molecules. Recent studies demonstrated that such models can be applied to designing drug candidates in early‐stage drug discovery such as hit generation and lead optimization. For example, Zhavoronkov et al. designed focused molecules as inhibitors against discoidin domain receptor family member 1, and their potency and physicochemical properties measured by experiments indeed satisfied conditions as lead candidates.^[^ ^6^ ^]^

The architectures of various deep generative models rely on molecular representations employed as input for molecular structures. Language models and variational autoencoders with simplified molecular input line entry system (SMILES) molecular representation have been widely used.^[^ ^7^, ^8^, ^9^, ^10^, ^11^, ^12^, ^13^ ^]^ The language models are trained to construct new SMILES strings by sequentially adding new characters to a given piece of SMILES string. Then, the models can generate novel molecules not in the training set by exploring the chemical space learned from the training. Variational autoencoder (VAE) is also a popular architecture.^[^ ^11^ ^]^ In VAE, the encoder converts a given molecular representation as input to an embedding vector in the latent space, and the decoder recovers the original molecule from the latent vector. After training, the VAE model can generate new molecules by decoding latent vectors sampled from the resulting latent space. The controlled sampling of latent vectors allows us to manipulate the structural diversity and similarity of the generated molecules. It is also possible to control the molecular properties of the generated molecules by adopting additional training strategies such as reinforcement learning,^[^ ^14^, ^15^, ^16^, ^17^, ^18^ ^]^ transfer learning,^[^ ^7^ ^]^ conditional generation,^[^ ^19^, ^20^, ^21^ ^]^ and Bayesian optimization.^[^ ^11^, ^22^, ^23^, ^24^, ^25^ ^]^ Other kinds of deep generative models utilizing molecular graphs have been proposed.^[^ ^26^, ^27^ ^]^ The graph‐based models can be readily modified for specialized purposes such as scaffold‐based design and 3D linker design through specifically controlling molecular graph structures because their nodes and edges directly correspond to atoms and chemical bonds.^[^ ^28^, ^29^, ^30^, ^31^, ^32^, ^33^, ^34^ ^]^ Lim et al.^[^ ^30^ ^]^ and Li et al.^[^ ^31^ ^]^ proposed scaffold‐based molecule generation algorithms for early stage drug discovery such as hit‐to‐lead and lead optimization in which molecular structures can be adjusted without changing a designated core scaffold. Imrie et al. proposed a linker generation model while conserving given fragments and their coordinates, which can assist to combine small molecules in fragment‐based drug discovery.^[^ ^32^ ^]^

Despite the promising results of previous models, their common design strategy, which sequentially adds atoms and bonds in graph‐based models or characters in SMILES‐based models, would be chemically less intuitive. Also, many molecules generated via the previous models have low synthesizability.^[^ ^35^ ^]^ Human experts mostly perceive a molecule as a connected set of chemical building blocks rather than a simple assembly of atoms. In terms of designing molecules, this conceptual perception is more practical because molecular properties are finely tuned by tailoring those building blocks. It is also advantageous in considering the synthetic feasibility of generated molecules. The direct consideration of synthesizability can be done, for example, by preparing synthetically accessible building blocks using known reaction templates and letting the model learn the implied synthetic validity from the resulting data.

In this regard, we propose a novel deep generative model for molecular generation, namely building block‐based autoregressive generative model (BBAR) which aims to design new molecules with target properties by sequentially adding building blocks to any given starting molecule. Our building block‐based molecule generation is theoretically in line with the commonly used concepts such as synthons and synthetic equivalents in retrosynthesis.^[^ ^36^ ^]^ In a training phase, the model learns to recover original molecules by adding building blocks to an arbitrarily given core structure. In a generation phase, it predicts a possible building block and corresponding atom pairs for making a bond between the building block and the core structure. At the end, a novel molecule with desired properties can be obtained by repeating the process.

We expect that the sequential addition of building blocks also helps the model learn how each building block affects the molecular properties, rather than simply memorizing the relationship between whole molecular structures and their properties in an end‐to‐end fashion. That is, the model can learn how to select appropriate building blocks and bind them with given core molecules to achieve target properties. Moreover, learning the contribution of each building block to target properties can encourage the model to produce novel molecules even with rare property values in the training set. In this perspective, the sequential addition of molecular building blocks is more beneficial than the sequential addition of atoms for learning the structure–property relationship with high generalization ability, because molecular properties are more correlated with functional molecular substructures than individual atoms.

To our best knowledge, there are a few fragment‐based molecular generative models, though the definition of fragments in those works is different from the building block in this work. Podda et al. proposed a language model which sequentially generates fragments and combining them into a single molecule.^[^ ^37^ ^]^ They could achieve the high validity and uniqueness of generated molecules. Chen et al. proposed a deep generative model for molecule optimization via one fragment modification.^[^ ^38^ ^]^ Yang et al. developed a reinforcement learning model that sequentially adds fragments to a given core molecule to improve the binding affinity of the resulting molecule to a target protein.^[^ ^39^ ^]^ The example study in the work showed a possibility of designing potential drug candidates with strong binding to the target. Despite the conceptual advance of these models, they have fundamental limitations in dealing with diverse fragments. Yang et al. sampled fragments from a predefined library which contains only 66 fragments. Podda et al. explicitly considered only a small number of frequent molecular fragments in a dataset. Furthermore, these models cannot accept novel fragments that are not in the training set, because they used fixed libraries. Chen et al. solved the limitations by representing fragments with latent vectors and searching fragments in the resulting latent space.^[^ ^3^ ^]^ However, sampling fragments from the latent space does not guarantee the synthetic accessibility of generated molecules especially when the fragments are not readily available.

It is suspected that formulating the building block selection problem from a library into a classification task may provoke such a limitation with the following problems. First, representing the whole set of building blocks as a single vector and training the classification model are computationally inefficient unless using a small number of selected building blocks. Previous fragment‐based models reduced the computational burdens by using a fixed size of restricted building block library.^[^ ^33^, ^37^, ^39^ ^]^ However, the use of a small number of building blocks reduces the diversity of generated molecules. Second, the model must be retrained whenever a new building block is added to the library. We resolve these problems by splitting the building block selection process into two steps. We first sample a building block randomly from a predefined library. Then, we determine whether the sampled building block will be added to a given core molecule, which can be done using a deep neural network that predicts the probability of connecting between the given molecule and the building block. The neural network can take any building blocks as an embedding vector obtained by encoding the molecules using another deep neural network, so one can add new building blocks in the library without retraining the model. This strategy allows us to handle an unlimited number of building blocks in theory while maintaining high computational efficiency.

## 2 Experimental Section

Here, the goal is to generate functional molecules for a specific purpose by sequentially adding building blocks to any given core molecule as input until satisfying desired properties. To this end, the model learned to reproduce the original molecule by sequentially adding building blocks to a given scaffold in an auto‐regressive manner. The process was similar to the language model in natural language processing.

**Figure 1:**
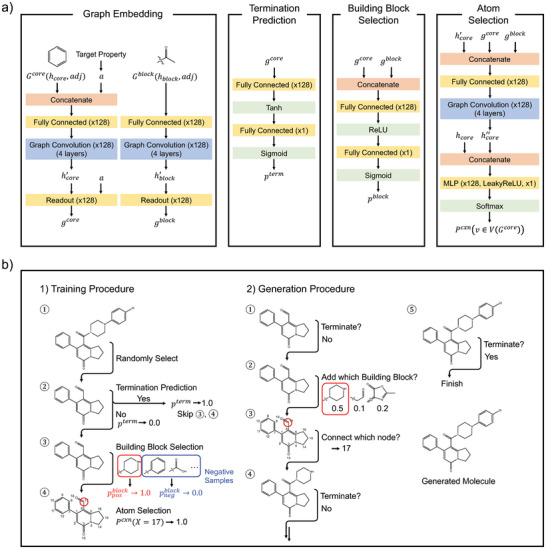
a) The schematic representation of the model. b) The training procedure (1) and the sampling procedure of the model (2).

This model has three sub‐modules: a building block selection module, an atom selection module, and a termination prediction module. The building block selection module predicted an appropriate building block to be added. The atom selection module found an atom pair for making a bond between the predicted building block and the core molecule: one from the predicted building block and the other from the core molecule. The termination prediction module determined whether the generation process should be terminated or repeated. **Figure** **1** schematically shows the model architecture and the process of the training and generation. The details of each sub‐module and the processes are described in the following subsections. All source code and data can be available at https://github.com/jaechang‐hits/BBAR‐pytorch.

### 2.1 Dataset

An important prerequisite for developing building block‐based deep generative models was the preparation of an appropriate building block library. The library should contain all building blocks of the molecules in the training set, preferably many molecules, so that the model could learn chemical diversity. There would be various definitions of building blocks such as BRICS,^[^ ^40^ ^]^ RECAP,^[^ ^41^ ^]^ Bemis–Murcko,^[^ ^42^ ^]^ and so on. There would be various definitions of building blocks. One straightforward way was to prepare building blocks by decomposing whole molecules using fragmentation methods such as BRICS, RECAP, Bemis‐Murcko, and so on. In principle, the proposed model works with any definition. In this work, the BRICS decomposition was adopted. The algorithm of the BRICS decomposition was breaking the covalent bonds which corresponded to predefined SMILES arbitrary target specification (SMARTS) strings. These SMARTS strings corresponded to synthetically accessible bonds, so BRICS decomposition was similar to retrosynthesis. The resulting building blocks had labels on their atoms indicating whether the formation of chemical bonds at the atom was possible. These labels helped the model generate synthetically more feasible molecules in the generation process. 249 456 molecules were divided from the ZINC15 dataset^[^ ^43^ ^]^ into the training set of 220 011, the validation set of 24 445, and the test set of 5000 molecules. The BRICS decomposition was applied to those molecules, resulting in 42 255 unique building blocks.

### 2.2 Building Block Selection

To handle many building blocks efficiently in the dataset, the building block selection task was formulated into a matching problem of two molecular graphs for connection. The selection module took two molecular graphs $G^{\mathrm{core}}=\left(V^{\mathrm{core}},E^{\mathrm{core}}\right)$, $G^{\mathrm{block}}=\left(V^{\mathrm{block}},E^{\mathrm{block}}\right)$ and *a* as input, where *V* and *E* denote a set of nodes (or atoms) and edges (or bonds) in a given graph (molecule), and *a* denotes condition. The condition vector *a* target properties, such as molecular weight, in the core molecule, and a null vector in the building blocks. The target properties were the properties of the original molecule in the training process. Then module predicted the probability of binding *G* ^core^ and *G* ^block^. Each graph *G* had nodes $v_{i}\in V$ and edges $e_{\mathrm{ij}}\in E$, where *i* and *j* denote node indices. In this work, *G* ^core^ and *G* ^block^ were the molecular graph of a core molecule given as input or from the previous step and a building block sampled randomly from the building block library, respectively. A revised version of the graph convolution network proposed by Kipf et al. was used^[^ ^44^ ^]^ and the modified model was defined as follows:

(1) $$h_{i}^{\prime }=\phi_{1}\left(h_{i}∥a\right)$$

(2) $$h_{i}^{\prime \prime }=ReLU\left(\sum_{j\in N_{i}}\phi_{2}\left(h_{j}^{\prime }\right)\right)$$

(3) $$c_{i}=\sigma \left(\phi_{3}\left(h_{i}^{\prime \prime }∥h_{i}^{\prime }\right)\right)$$

(4) $$h_{i}^{\prime \prime \prime }=c_{i}h_{i}^{\prime }+\left(1-c_{i}\right)h_{i}^{\prime \prime }$$

where $h_{i}$ is a *n*‐dimensional embedding vector of $v_{i}$, RELU is a ReLU activation function, σ is a sigmoid activation function, $N_{i}$ is the neighboring nodes of the *i*th node, and ϕ_1_, ϕ_2_, and ϕ_3_ are fully connected layers, respectively. The output embedding vector $h_{i}^{\prime \prime \prime }$ of a graph convolution layer was used as an input embedding vector of the next graph convolution layer ($h_{i}:=h_{i}^{\prime \prime \prime }$). The purpose of Equation (1) was to embed a condition vector with $h_{i}$ into a vector and to achieve enough expressive power by adopting a linear layer. Then, the model learned the joint probability distribution of the given molecule and its properties. After applying the graph convolution layers several times, a graph vector *g* of *G* was obtained from the weighted summation of the final embedding vectors;

(5) $$g_{\mathrm{sum}}=\sum_{i}\sigma \left(\phi_{4}\left(h_{i}\right)\right)\phi_{5}\left(h_{i}\right)$$

(6) $$g=\phi_{6}\left(g_{\mathrm{sum}}∥a\right)$$

where ϕ_4_ , ϕ_5_, and ϕ_6_ are fully connected layers. By applying the graph convolution layers to each of *G* ^core^ and *G* ^block^, the two respective graph vectors *g* ^core^ and *g* ^block^ were obtained. To train the conditional model, molecular properties were incorporated into the condition vectors of *G* ^core^. The probability value for building block selection *p* ^block^ was evaluated as a function of *g* ^core^ and *g* ^block^ as follows.

(7) $$p^{\mathrm{block}}=\sigma \left(\phi_{7}\left(\left(g^{\mathrm{core}}∥g^{\mathrm{block}}\right)\right)\right)$$

where ϕ_7_ is a neural network made of fully connected layers.

The training dataset made by the BRICS decomposition intrinsically included only positive samples, where “positive” means that building blocks of a molecule could be properly added to the other building blocks of the same molecule prepared by the decomposition method. To make the model select such building blocks over others, it was also needed to train the model with namely the negative samples which were unlikely to be added. To prepare the negative samples for each positive sample, ten building blocks proportional to their occurrence to the power of 3/4 as proposed in ref. [45] was randomly chosen. This training strategy, called the negative sampling, was often used in the Word2Vec model in the natural language processing.^[^ ^45^ ^]^ The model was trained to predict the building block probability *p* ^block^ as 1.0 for the positive samples and 0.0 for the negative samples. The binary cross‐entropy loss was used for this task. The objective function *J*_block_ of the building block selection module is as follows:

(8) $$J_{\mathrm{block}}=\log p_{\mathrm{pos}}^{\mathrm{block}}+\frac{1}{N}\sum_{i}^{N}\log\left(1-p_{\mathrm{neg},i}^{\mathrm{block}}\right)$$

where *N* is the number of negative samples per each positive sample, and $p_{\mathrm{pos}}^{\mathrm{block}}$ and $p_{\mathrm{neg}}^{\mathrm{block}}$ are predicted probability values for positive samples and negative samples, respectively. In this research, *N* is set to 10.

### 2.3 Atom Selection

The atom selection module predicted the bonding probability between $v_{i}^{\mathrm{core}}\in V\left(G^{\mathrm{core}}\right)$ and $v_{j}^{\mathrm{block}}\in V\left(G^{\mathrm{block}}\right)$. The atom for the bonding in the selected building block *G* ^block^ was already labeled when it was prepared by the BRICS decomposition. Hence, the need was to choose the counterpart atom from *G* ^core^. The atom selection module accepted output node embedding vectors from the building block selection module and applied the graph convolution layers to them. After applying the graph convolution layers, the connection probability $p_{i}^{\mathrm{cxn}}$ of $v_{i}^{\mathrm{core}}\in V\left(G^{\mathrm{core}}\right)$ was calculated using fully connected layers with a softmax layer at the end. The model was trained to predict $p_{i}^{\mathrm{cxn}}$ as 1 for a positive atom and 0 for negative atoms. The cross‐entropy loss for this task was used. The objective function *J*_selection_ is as follows

(9) $$J_{\mathrm{selection}}=\frac{1}{|V(G^{\mathrm{core}})|}\sum_{v_{i}\in V(G^{\mathrm{core}})}y_{i}^{\mathrm{cxn}}\log p_{i}^{\mathrm{cxn}}$$

where $y_{i}^{\mathrm{cxn}}$ is the true label of $v_{i}$ indicating whether the atom is positive or negative.

### 2.4 Termination Prediction

The termination prediction module predicted the probability of terminating the generation process. The module produced a graph vector *g* ^core^ by applying the graph convolution layers (Equations (1)–(4)) and weighted summation of embedding vectors (Equations (5) and (6)). Then, the termination probability can be evaluated by applying fully connected layers ϕ_8_ with the sigmoid activation function to the graph vector *g* ^core^, which is given by

(10) $$p^{\mathrm{term}}=\sigma \left(\phi_{8}\left(g^{\mathrm{core}}\right)\right)$$

In the training process, the module was trained to predict the termination probability *p* ^term^ as 1.0 when the original molecules were recovered and otherwise as 0.0. The objective function *J*_termination_ is given by

(11) $$J_{\mathrm{termination}}=y^{\mathrm{term}}\log p^{\mathrm{term}}+\left(1-y^{\mathrm{term}}\right)\log\left(1-p^{\mathrm{term}}\right),$$

where *y* ^term^ is the termination label.

### 2.5 Molecular Generation after Training the Model

The model accepted a starting molecule as input to generate a larger molecule by adding building blocks to it. If the starting molecule was not given, a building block was randomly selected from the building block library as a starting molecule. The conditional molecule generation additionally needed target properties as input. The generation process began to predict the termination probability of the starting molecule. Then, the termination sign was sampled in proportion to the termination probability given by Equation (10). If not terminating, the model executed the building block selection and the atom selection modules subsequently. In the building block selection step, the model randomly samples a set of building blocks proportional to their populations in the training. This stochastic sampling enhanced the efficiency of the generation process. The number of building blocks in each sampling was a hyper‐parameter, and here 2000 building blocks were sampled at each time. After sampling the building blocks, the building block selection module predicted the matching probability of every building block for addition and stochastically selected one of them in proportion to its predicted probability. Then, the atom selection module predicted the connection probability of all possible atoms in the starting molecule. One atom was stochastically chosen in proportion to the predicted probability. Finally, the labeled atom of the building block was connected with the chosen atom. These procedures were repeated until the termination sign was on. Figure 1a shows the model structure, and the hyper‐parameters are summarized in Section S1, Supporting Information.

## 3 Results and Discussion

### 3.1 Controlling Molecular Properties of Generated Molecules

One criterion of assessing the performance of deep generative models for molecule generation is comparing the designated target property and the actual property of generated molecules. For this purpose, we trained five instances of the model with four properties: molecular weight (MW), simple log water‐octanol partition coefficient (LogP),^[^ ^46^ ^]^ topological polar surface area (TPSA),^[^ ^47^ ^]^ and quantitative estimation of drug‐likeness (QED).^[^ ^48^ ^]^ All these properties were calculated by RDKit.^[^ ^49^ ^]^ After training, we obtained 100 core structures from the test set that are not included in the training and validation set and generated 100 molecules for each core structure. For the property control task, we only tested the molecule generation starting from given core structures because it is more challenging than de novo design due to the constraint imposed by the fixed starting molecules. We repeated the process for the four target properties. Because it is unphysical to reduce MW or TPSA by adding building blocks, we randomly chose 100 molecules where MWs are smaller than 200 and TPSA values are small than 40.

**Figure 2:**
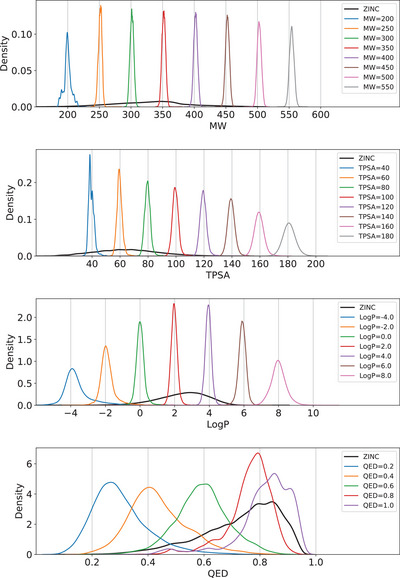
The property distributions of the molecules generated by the model conditioned on LogP, MW, TPSA, and QED, respectively. The black line indicates the property distribution of the training set, while the color lines denote those of the generated molecules with various target properties.

**Figure** **2** shows the property distribution of the generated molecules with different target properties. For the case of LogP, MW, and TPSA, their peak positions are at the target property, indicating that the model successfully learned the structure–property relationship in terms of controlling the molecular properties. Even in regions where training data points are sparse, the distribution is as sharp as in data‐rich regions. It was possible because the model learns the contribution of each building block to the target molecular properties instead of learning the end‐to‐end mapping between the whole molecular structure and its property, as mentioned in the introduction. Moreover, our model can control more complex properties such as QED. For instance, the top five QED scores of 10 000 generated molecules whose target QED value was set to 1.0 were 0.948. The value of 0.948 is equal to the highest value reported in the QED maximization task using optimization models such as reinforcement models and Bayesian optimization.^[^ ^18^, ^25^, ^50^, ^51^, ^52^, ^53^ ^]^ This result shows the feasibility of that our conditional generation model can also be used for optimization if the target value has a well‐defined range like QED. The full results for validity, uniqueness, and average property are available in Section S3, Supporting Information.

Also, it is meaningful to compare our model with the previous scaffold‐based GGM proposed by Lim et al.,^[^ ^30^ ^]^ which generates new molecules by sequentially adding atoms and bonds to a given scaffold. For comparison, we calculated the validity, uniqueness, and average property of the generated molecules. The exact definitions of the validity and the uniqueness can be found in ref. [54]. These metrics indicate not only the efficiency of molecule generation but also the quality of learned distributions. The average property shows the mean value and the standard deviation of calculated properties. We estimated the performance of the scaffold‐based GGM for the four conditions using the same dataset. **Table** **1** shows that our model outperformed the previous scaffold based model in all aspects.

**Table 1:** Comparison of the performance in the conditional generation task. The validity, uniqueness, and average property of the conditional generation

	Ours (BBAR)			Lim et al.		
Target	Validity	Uniqueness	Average property	Validity	Uniqueness	Average property
MW = 250	1.000	0.808	** 251.217 ± 2.925**	0.981	0.431	248.358 ± 4.103
TPSA = 140	1.000	0.999	**139.428 ± 4.611**	0.904	0.970	136.729 ± 7.654
LogP = 2.0	1.000	0.960	**1.970 ± 0.211**	0.973	0.763	2.188 ± 0.657
QED = 1.0	1.000	0.871	**0.818 ± 0.099**	0.961	0.761	0.795 ± 0.097

**Figure 3:**
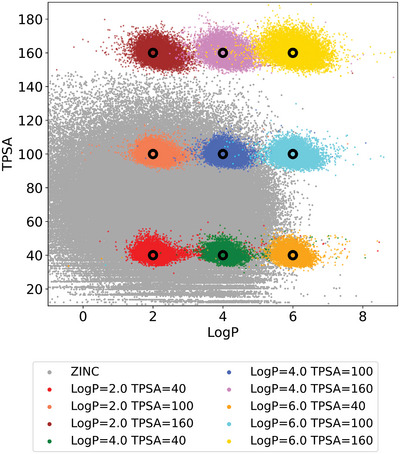
The LogP and TPSA values of the molecules generated by the model conditioned on both LogP and TPSA. The gray dots are for the training set, while the color dots are for the generated molecules. We used RDKit to calculate the LogP and TPSA of the molecules.

We further tested whether the model can control multiple molecular properties of generated molecules. For that purpose, we trained the instance of the model with LogP and TPSA, and generated 100 molecules for each of the same 100 starting molecules used in the previous experiment. **Figure** **3** shows the property distributions of the molecules as a function of the target values. The gray points indicate molecules in the training set. Though the distributions are more spread out than the case of the single property control, their peak positions are at the target values. It supports that the model can correctly learn the structure‐multiple properties relationship. In addition, the model performs well for the extreme targeted values around TPSA of 160 and LogP of 6, though the training data is rare around these target values as depicted in Figure 3.

### 3.2 Generalization of Model on Unseen Building Blocks

**Figure 4:**
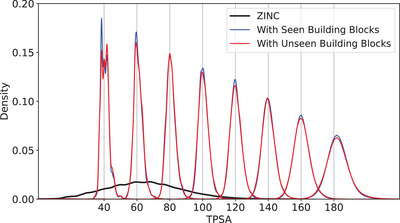
The TPSA distribution of the generated molecules with the seen (blue line) and unseen (red line) building blocks.

One distinctive advantage of our model is that any building blocks not necessarily in the training set can be used for molecule generation because the model takes the embedding vectors of building blocks converted by a neural network as input. To verify the feasibility, we tested the model whether it can generate new molecules with target properties at a high success rate by adding unseen building blocks. We first excluded randomly chosen 14 085 building blocks out of 42 255 building blocks during the training process and used them as the unseen building blocks in the generation process. The generation process is identical to that of the previous experiment described in Section 3.1 except using only the excluded building blocks for addition. After training the model conditioned on TPSA, we generated 100 molecules using the same 100 starting molecules used in the previous experiments but with the excluded building blocks. **Figure** **4** shows the result. The red and blue lines show the property distributions of the generated molecules with the seen and unseen building blocks, respectively. For both cases, the distribution peaks place at the respective target properties, which proves that the model equally well performs with unseen building blocks.

In the real‐world application of molecule generation, it is likely to impose certain constraints in molecule design. For example, in drug discovery, medicinal chemists may want to add a hydrophobic fragment or linker to a core structure according to their structure–activity relationship analysis. The atom addition strategy needs several steps to meet the condition, leading to a low success rate. In contrast, our model can readily offer a practical strategy. For instance, we can force the model to add only hydrophilic building blocks by providing only hydrophilic building blocks for addition. Our model is particularly suitable for this purpose because it works well with unseen building blocks.

**Figure 5:**
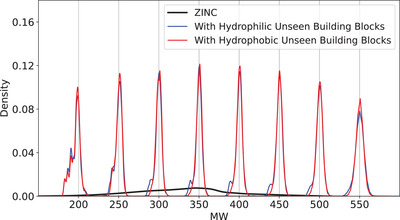
The MW distribution of the generated molecules with the hydrophobic (blue lines) and hydrophilic (red lines) unseen building block.

**Figure 6:**
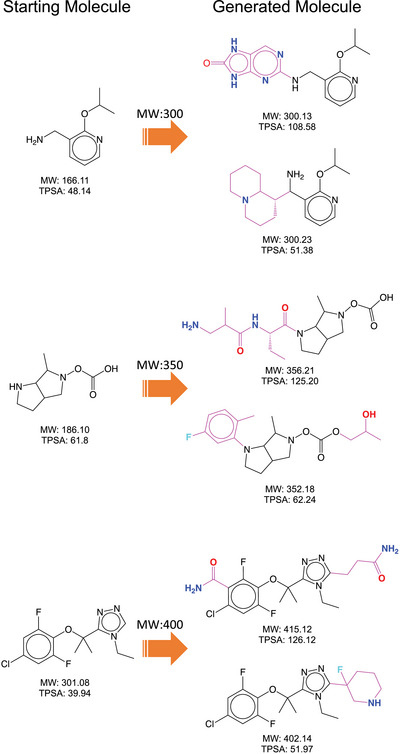
The examples of the generated molecules with the hydrophobic and hydrophilic building blocks. The molecules on the left side are starting molecules. The top and bottom molecules on the right side were obtained from the molecule generation with hydrophilic and hydrophobic building blocks, respectively. The molecules generated with the hydrophilic building blocks include more nitrogen and oxygen atoms. In contrast, the molecules generated with the hydrophobic building blocks have more hydrocarbons.

For demonstration, we prepared two sets containing unseen hydrophilic and hydrophobic building blocks, respectively. The hydrophobic building block set is made of 2000 building blocks with relatively low TPSA values, which appear more than five times in the dataset. For the hydrophilic building block set, we chose the top 2000 building blocks in terms of the TPSA value also with more than five times occurrences in the dataset. **Figure** **5** shows the MW distribution of the molecules generated by the model conditioned on MW when we design new molecules with the hydrophilic and hydrophobic building block sets, respectively. The blue and red lines indicate the generated molecules with hydrophilic and hydrophobic building blocks, respectively. Like the previous result shown in Figure 2, each distribution peak locates at the respective target value for both hydrophilic and hydrophobic building block sets. **Figure** **6** shows the several examples of the generated molecules and their starting molecules. The molecules generated with the hydrophilic building blocks include more nitrogen and oxygen atoms as intended, whereas those generated with the hydrophobic building block have more hydrocarbons.

### 3.3 Application to Drug Design: Demonstration of Designing a Novel Inhibitor against the 3CL Protease of SARS‐CoV‐2

**Figure 7:**
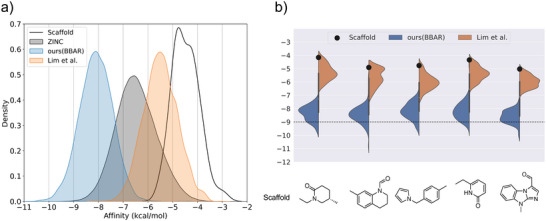
The docking score distributions of the training set (ZINC), the start core structures (scaffold), and the molecules generated by our model (BBAR) and the scaffold‐based generative model proposed by Lim et al.^[^ ^30^ ^]^ a) The total distributions of 10 000 molecules generated from 100 core structures. 100 molecules were generated from each core. b) The distributions of molecules generated from the five randomly chosen core structures which are shown below the respective curve.

As a real‐world application, we applied our model to designing novel inhibitors against the 3CL protease of SARS‐CoV‐2. The 3CL protease is one of the most widely studied biological targets for the development of anti‐SARS‐CoV‐2 drug. The objective of this experiment is to let the model learn the relationship between molecular structures and their binding affinities against the target protein. In other words, the model learns which building blocks should be added to a given molecule to increase the binding affinity. For model training, we used the same dataset in the previous experiment, which contains the training set of 220 011, the validation set of 24 445, and the test set of 5000 molecules. After training, this model produces molecules that are more likely to bind to the target. Considering more than millions of learnable parameters in the model, we need a large amount of data for training. However, experimental data is not sufficient. Instead, we used simulation data as a demonstration. We performed docking calculations with the same molecular library used in the previous experiments in Section 3.1 and the 3CL protease of SARS‐CoV‐2 whose PDB id is 7L13. We used Smina,^[^ ^55^ ^]^ the fork of Autodock Vina,^[^ ^56^ ^]^ for the docking calculations with the default setting. The initial conformers of the molecules in the library were obtained by the universal force field^[^ ^57^ ^]^ calculation of RDKit. The calculated docking scores have used as the labeled data for conditional generation. Note that the lower the docking score, the higher the binding affinity, due to the negative sign of the docking score. **Figure** **7** shows the docking score distribution of the molecules in the training set. The molecules with docking scores lower than −9.0 kcal mol^−1^ are in the top 0.049% of the training set.

The generation started with the randomly chosen building blocks of test molecules, yielding 10 000 molecules with a target docking score of −10.0 kcal mol^−1^. Figure 7 compares the docking score distributions of molecules generated by our model and the scaffold‐based GGM model with that of the training set. The distribution of the generated molecules substantially shifts toward lower docking scores from that of the training set. As a result, the portion of molecules with docking scores lower than −9.0 kcal mol^−1^ increased about 170 times from 0.049% to 8.55%, which manifests the feasibility of our model for practical applications to drug design. Since each building block represents unique chemical functionality, it is easier for our model to create additional protein‐ligand interaction than the atom‐based model does.

We also evaluated the chemical diversity of the generated molecules with high binding affinities in terms of docking scores. The definition of chemical diversity is the average pairwise Tanimoto distance between Morgan fingerprints^[^ ^58^ ^]^ of the molecules.^[^ ^5^ ^]^ The high diversity value means that the molecular set includes more diverse chemical structures in terms of molecular similarity. The diversity of the generated molecules and the training molecules with high binding affinities are 0.871 and 0.858. It means that the generated molecules are as diverse as the training set that includes a variety of chemical structures, and the model produces various chemical structures, which are not simply the same core with slightly different small residues. In addition, the novelty value of the generated molecules is 1.0, meaning that the generated molecules are unique, not in the training set.

Despite the success of designing molecules with the target docking score to some extents, the distribution peak deviates significantly from the target value, which is contrast to the previous cases of targeting MW, LogP, and TPSA. MW, LogP, and TPSA can be determined solely by the properties of each building block for addition. In contrast, the docking score is determined not by the building block itself but by its interaction with the target protein. Even a single building block addition may change the entire interaction mode of a molecule by altering its binding pose. Since we did not consider the target protein structure explicitly in the generation process, the model needs to learn all possible interaction changes upon the building block addition, which is not straightforward. Therefore, the success rate of designing molecules with a target docking core is substantially lower than that of the previous cases.

## 4 Conclusion

Chemical building blocks such as functional groups are closely related to molecular properties and synthetic accessibility. Thus, building block‐based molecular design can facilitate better controlling molecular properties with high synthetic accessibility. Here, we proposed a novel deep generative model that aims to design desired molecules by assembling retrosynthetically prepared chemical building blocks. It generates new molecules by sequentially adding building blocks to a given starting molecule. Dealing with many building blocks including unseen is essential for a high diversity of the resulting molecules. This cannot be achieved if representing building blocks with a fixed vector and applying a classifier to the vector. Instead, we devised a model that predicts the bonding probability of any two molecules: one from the given core molecule and the other from a building block library. Therefore, the model does not limit the number of building blocks in the library. Furthermore, the model takes the embedding vector of building blocks encoded by a deep neural network as input, which enables the model to accept unseen building blocks after training. This strategy leads to a high generalization ability of the model in term of building block diversity. Building blocks for training and generation were prepared using the BRICS decomposition method which explicitly takes into account synthetic feasibility when decomposing complete molecules. Hence, the model can implicitly learn synthetic accessibility from the data prepared as such.

Our model consists of three modules: building block selection, atom selection, and termination prediction modules. The building block selection module evaluates the bonding probability between a given molecule and a building block. Then, the atom selection module finds out the most probable atom pairs for making a chemical bond between the two molecules. Finally, the termination prediction gives the probability of terminating the molecule generation process.

We assessed the model performance in various tasks. First, the model was able to control the molecular weight, topological polar surface area, LogP, and QED while generating molecules with a high success rate. Such high performance retained with unseen core molecules or unseen building blocks. This result supports that the model achieved a good generalization ability to some extents rather than simply memorizing the hidden pattern of the training set. This generalization ability comes from learning how to tune molecular properties by adding appropriate building blocks in a step‐wise manner. For instance, the model can design molecules with target properties out of the distribution of the training set. We also demonstrated that target properties can be achieved by adding only hydrophobic or hydrophilic building blocks. These cases cannot be done with previous models that generate molecules by adding atoms and bonds. As a practical application, we successfully designed candidate inhibitors showing high binding affinities against the 3CL protease of SARS‐CoV‐2 in terms of docking score. We believe that our building block‐based deep generative model paves a practical way of molecular design with high synthesizability for various chemical applications such as drug discovery.

## Conflict of Interest

The authors declare no conflict of interest.

## Supporting information

Supporting Information

## Data Availability

The data that support the findings of this study are openly available in ZINC 15 – Ligand Discovery for Everyone at https://doi.org/10.1021/acs.jcim.5b00559, reference number 43.
