# Supplementary material for: Molecular Generative Model via Retrosynthetically Prepared Chemical Building Block Assembly
Source: Adv Sci (Weinh). 2023 Jan 3;10(8):2206674. doi: 10.1002/advs.202206674 (PMC10015872; doi:10.1002/advs.202206674)
Supplement: Supplementary file 1 — Supporting Information [file ADVS-10-2206674-s001.pdf]

Supporting Information

Molecular generative model via retrosynthetically prepared chemical building block assembly

Seonghwan Seo[1,2] | Jaechang Lim[1*] | Woo Youn Kim[1,2,3*]

[1]HITS incorporation, 124, Teheran-ro, Gangnam-gu, Seoul 06234, Republic of Korea. Email: jaechang@hits.ai

[2]Department of Chemistry, KAIST, 291 Daehak-ro, Yuseong-gu, Daejeon 34141, Republic of Korea. Email: wooyoun@kaist.ac.kr

[3]AI Institute, KAIST, 291 Daehak-ro, Yuseong-gu, Daejeon 34141, Republic of Korea. Email: wooyoun@kaist.ac.kr

A | HYPERPARAMETERS OF THE MODELS

TABLE 1 The hyper-parameter of the model training.

Hidden dimension	Learning rate	Batch size	Number of negative Samples
128	0.001	128	10

B | RECONSTRUCTION PERFORMANCE

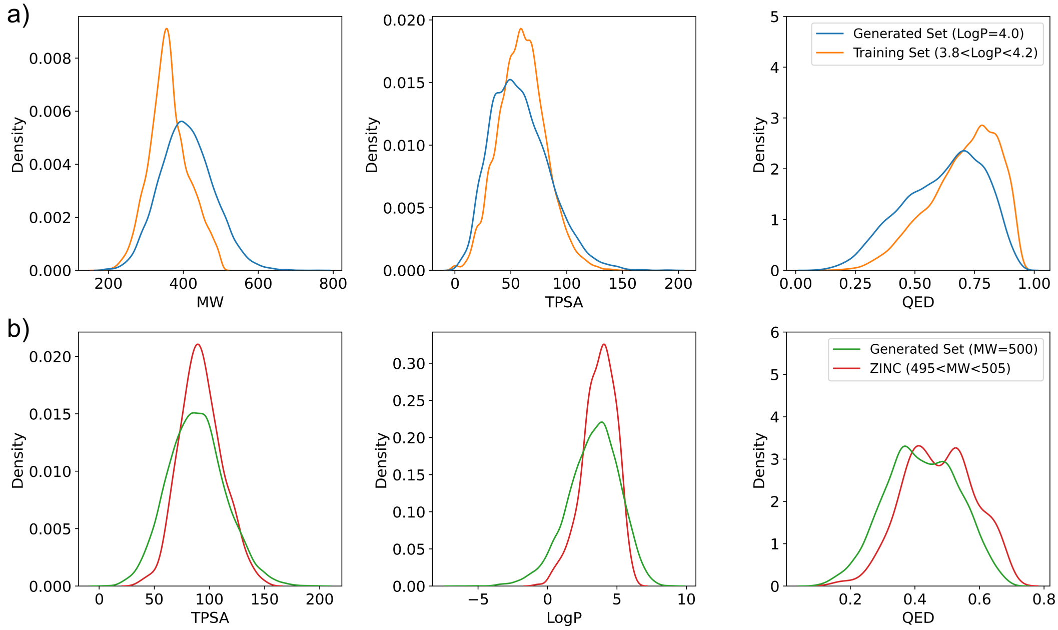

FIGURE 1 Comparison of the distributions of the training set (or ZINC) molecules and the generated molecules with a specific target condition. a) The distributions of MW, TPSA, and QED when the target condition in the generation was set as LogP = 4.0. b) The distributions of TPSA, LogP, and QED when the target condition in the generation was set as MW = 500.

C | RESULT OF CONDITIONAL GENERATION

TABLE 2 The validity, uniqueness, novelty, diversity, and average property of molecules generated with a specific target condition given in the second column. The average property means the mean value of the computed properties of the generated molecules. The diversity was calculated for each core structure. The Smina score means the docking score of each molecule against 3CL protease computed by Smina.

Property	Target	Validity	Uniqueness	Novelty	Diversity	Average Property
MW	200	1.000	0.251	1.000	0.467	199.606 ± 5.813
	250	1.000	0.808	1.000	0.689	251.217 ± 2.925
	300	1.000	0.975	1.000	0.732	301.377 ± 2.957
	350	1.000	0.996	1.000	0.759	351.782 ± 2.944
	400	1.000	1.000	1.000	0.776	402.164 ± 3.017
	450	1.000	1.000	1.000	0.788	452.459 ± 3.157
	500	1.000	1.000	1.000	0.797	502.784 ± 3.366
	550	1.000	1.000	1.000	0.809	554.188 ± 3.668
TPSA	40	1.000	0.956	1.000	0.732	39.476 ± 2.076
	60	1.000	0.993	1.000	0.761	59.719 ± 2.080
	80	1.000	0.997	1.000	0.775	79.668 ± 2.207
	100	1.000	0.999	1.000	0.788	99.624 ± 2.379
	120	1.000	1.000	1.000	0.797	119.551 ± 2.482
	140	1.000	0.999	1.000	0.804	139.428 ± 4.611
	160	1.000	0.997	1.000	0.810	158.586 ± 11.394
	180	1.000	0.994	1.000	0.817	179.150 ± 17.238
LogP	-4.0	1.000	0.998	1.000	0.789	-3.638 ± 0.795
	-2.0	1.000	0.993	1.000	0.782	-1.872 ± 0.473
	0.0	1.000	0.977	1.000	0.767	0.025 ± 0.277
	2.0	1.000	0.960	1.000	0.757	1.970 ± 0.211
	4.0	1.000	0.993	1.000	0.758	3.944 ± 0.190
	6.0	1.000	1.000	1.000	0.774	5.901 ± 0.291
	8.0	1.000	0.996	1.000	0.800	7.949 ± 0.694
QED	0.2	1.000	0.999	1.000	0.796	0.301 ± 0.100
	0.4	1.000	0.988	1.000	0.784	0.437 ± 0.105
	0.6	1.000	0.960	1.000	0.763	0.594 ± 0.094
	0.8	1.000	0.984	1.000	0.720	0.763 ± 0.079
	1.0	1.000	0.871	1.000	0.705	0.818 ± 0.099
Smina Score (3CL)	-9.0	1.000	1.000	1.000	0.773	-7.839 ± 0.616

D | BENCHMARK STUDY

In this task, we chose 1,000 core structures from the test set whose MWs were lower than 175 and generated one molecule from each selected core. We used the median value of the property range given in the first row in Table 3 as the target property of generation.

TABLE 3 Comparison of the property conditioning performance of BBAR. The results of the other methods were obtained from ref [1]

Method	$-2.5 \leq$ LogP ≤ -2		$5 \leq$ LogP ≤ 5.5		$150 \leq$ MW ≤ 200		$500 \leq$ MW ≤ 550	
	Success	Diversity	Success	Diversity	Success	Diversity	Success	Diversity
ZINC	0.3%	0.919	1.3%	0.909	1.7%	0.938	0	-
JT-VAE[2]	11.3%	0.846	7.6%	0.907	0.7%	0.824	16.0%	0.898
ORGAN[3]	0	-	0.2%	**0.909**	15.1%	0.759	0.1%	0.907
GCPN[1]	**85.5**%	0.392	54.7%	0.855	76.1%	**0.921**	74.1%	**0.920**
BBAR	61.8%	**0.891**	**79.8**%	0.881	**100.0**%	0.915	**100.0**%	0.880

E | SAMPLES OF MOLECULES FOR 3CL PROTEASE

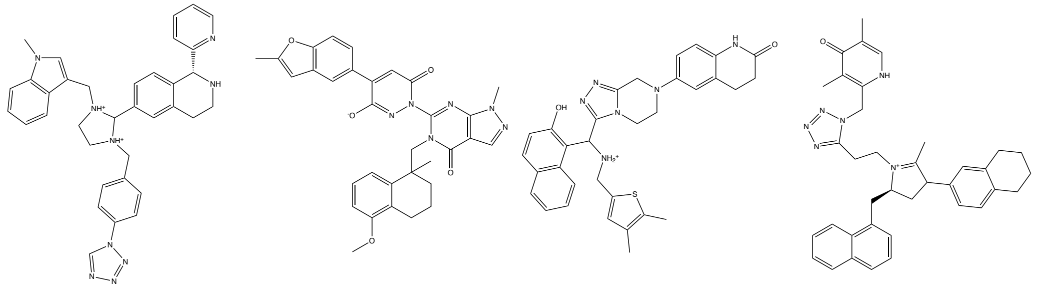

Smina Score: -10.335 Smina Score: -10.264 Smina Score: -10.235 Smina Score: -10.215

FIGURE 2 Samples of molecules with high Smina scores generated by our model (BBAR).

references

[1] You J, Liu B, Ying Z, Pande V, Leskovec J. Graph convolutional policy network for goal-directed molecular graph generation. Advances in neural information processing systems 2018;31.

[2] Jin W, Barzilay R, Jaakkola T. Junction Tree Variational Autoencoder for Molecular Graph Generation. In: Dy J, Krause A, editors. Proceedings of the 35th International Conference on Machine Learning, vol. 80 of Proceedings of Machine Learning Research Stockholmsmässan, Stockholm Sweden: PMLR; 2018. p. 2323–2332. http://proceedings.mlr.press/v80/jin18a.html.

[3] Guimaraes GL, Sanchez-Lengeling B, Outeiral C, Farias PLC, Aspuru-Guzik A. Objective-Reinforced Generative Adversarial Networks (ORGAN) for Sequence Generation Models. arxiv preprint:170510843 2017 may;http://arxiv.org/abs/1705.10843.
